# Assessing the Frequency, Prescribing Patterns, and Characteristics of Patients Receiving Drugs with Pharmacogenomic (PGx) Guidelines Through an EMR: Follow-Up Analysis 5 Years Later

**DOI:** 10.3390/pharmacy14020053

**Published:** 2026-03-25

**Authors:** George E. MacKinnon, Megan Mills, Ulrich Broeckel

**Affiliations:** 1 School of Pharmacy, Medical College of Wisconsin, Milwaukee, WI 53226, USA; ubroeckel@mcw.edu; 2 Department of Pharmacy, Froedtert and Medical College of Wisconsin, Milwaukee, WI 53226, USA; megan.mills@froedtert.com; 3 Department of Pediatrics, Medical College of Wisconsin, Milwaukee, WI 53226, USA; 4 RPRD Diagnostics LLC., Milwaukee, WI 53226, USA

**Keywords:** pharmacogenomic drugs, pharmacogenomic testing, electronic medical records, evidence-based guidelines, pharmacy, pharmacists

## Abstract

(1) Background: This follow-up retrospective analysis used electronic medical record (EMR) data from a health system to identify patients and medications prescribed in accordance with Clinical Pharmacogenetics Implementation Consortium (CPIC) guidelines. (2) Methods: This analysis included EMR data from a clinical research data warehouse encompassing 928,291 patients seen at an academic medical center between 2020 and 2024. The study evaluated 75 commercially available medications linked to 52 evidence-based CPIC pharmacogenomic (PGx) guidelines. (3) Results: Of the 928,291 patient encounters, 709,673 medication orders were recorded, with 416,621 patients (44.8%) prescribed at least 1 of the 75 CPIC-associated medications. This compares with 845,518 patients who had an encounter in 2015–2019 with 590,526 medication orders, and 335,849 (56.9%) patients had medication orders represented by CPIC-associated medications. One to three CPIC-associated medications accounted for 76.6% of patients in 2020–2024 compared to 75.6% in 2015–2019. (4) Conclusions: The findings demonstrate that the proportion of patients prescribed a CPIC-actionable medication remained just under half of those evaluated within a single institution’s EMR. About three-quarters of patients over the ten-year period had between one to three CPIC-associated medications identified, and the top five classes of medications remained the same in the two periods. This understanding of patient volume may help organizations as they begin to assess the implementation of PGx services.

## 1. Introduction

Pharmacogenetics (PGx) examines how genetic variations among individuals influence their responses to medications. The goal of PGx is to enable clinicians to choose the most effective therapies and dosing strategies while minimizing the risk of adverse drug effects [1,2,3,4]. As a significant building block to precision medicine, this includes pharmacogenomics.

Pharmacogenomic (PGx) factors can influence over half of the of prescriptions in the U.S., and nearly everyone carries at least one high-risk genetic variant that can affect drug response [3,5,6,7]. PGx testing is often considered for reasons such as toxicity, side effects, lack of efficacy, family history, or pre-emptive treatment [8]. The U.S. Food and Drug Administration (FDA) includes pharmacogenomic labeling for more than 200 medications to support safer prescribing, with some labels incorporating “Black Box” warnings for potentially life-threatening risks [9,10,11]. Examples of medications with actionable PGx information include warfarin and codeine, where labels indicate whether genetic testing is recommended before use [12].

The international group of scientific and clinical experts that compose the Clinical Pharmacogenetics Implementation Consortium (CPIC) offers evidence-based guidelines that help clinicians interpret pharmacogenomic test results and apply them to clinically actionable prescribing decisions for well-characterized gene–drug pairs. When the CPIC recommends pharmacogenomic testing, this is after an evidence review by a panel of scientific experts that shows there is substantial published scientific evidence indicating testing should be conducted to improve treatment outcomes and/or reduce harm to patients [5]. These guidelines focus on the most common and clinically actionable gene–drug interactions and are intended for implementation across diverse clinical practice settings. Patient-specific diplotypes are interpreted to generate corresponding clinical phenotypes and drug dosing recommendations [5]. The CPIC categorizes evidence into four levels (A–D), with level “A” representing strong or moderate evidence supporting the use of genetic information to guide prescribing decisions. In contrast, level “D” denotes limited published evidence, unclear clinical actionability, minimal mechanistic rationale, predominantly weak data, or substantial conflicting findings. As of 2025, the CPIC had published 29 pharmacogenomic guidelines encompassing 35 genes and 168 medications across diverse therapeutic categories [5].

Pharmacogenetic (PGx) testing can be performed either reactively or pre-emptively. In the U.S., most PGx tests are reactive, ordered by a single prescriber after a patient experiences an adverse drug effect or fails to respond to therapy. These tests often focus on a single gene–drug pair, limiting future usefulness for other prescribers. In contrast, pre-emptive PGx testing is conducted before a medication is selected to help prevent adverse effects and improve treatment efficacy [6,13,14]. Because a patient’s genotype remains stable over time, pre-emptive results can guide future prescribing as health conditions and medication needs evolve, as well as when prescribers change over the lifetime of a patient. As costs decline and more guidelines develop, pre-emptive PGx testing is a more cost-effective strategy, especially when performed early in life, providing valuable information throughout a patient’s lifetime [3,6,13].

Building on our previous retrospective analysis of EMR data from 2015 to 2019 at a tertiary integrated health system, we examined records from 2020 to 2024 to identify patterns in medication prescribing based on CPIC guidelines. During the 2015–2019 period, 845,518 patients had at least one clinical encounter, resulting in 590,526 medication orders [15]. Of these patients, 335,849 (56.9%) received one or more medications included in the 52 CPIC guidelines. These prescriptions were issued by 2803 providers and encompassed 42 commercially drugs, highlighting the scope of PGx-relevant prescribing within the health system [15].

Identifying the scope of patients who may benefit from pharmacogenomic testing can support the development of proactive testing strategies, targeted physician resources, and pharmacist-led workflows to improve clinical outcomes if a PGx program is adopted. The release of updated CPIC guidelines, along with new medications and gene recommendations since 2019, motivated this follow-up retrospective analysis using EMR data from the same tertiary integrated health system. The primary aim was to assess if and how prescribing patterns have shifted in response to the updated CPIC guidelines. Conducting two internal analyses over a ten-year span provides valuable insight for medical center decision makers in evaluating the potential scope and necessity of implementing PGx services.

## 2. Materials and Methods

Deidentified electronic medical record (EMR) data from the academic medical center were consolidated within the Clinical Data Warehouse. The academic medical center is an adult Level I Trauma Center, located in Milwaukee, Wisconsin. At the time of data extraction there were more than 2000 physicians with over 40,000 inpatient admissions and approximately 1.5 million outpatient visits across 45 health centers and clinics [16].

We evaluated prescribing records and demographic characteristics for 928,291 patients with at least one encounter in the five-year period from 2020 to 2024. Patient records accessed included both inpatient and outpatient encounters. The drug formulary was cross-referenced with RxNorm at the ingredient level to extract all medication orders corresponding to 75 drugs meeting CPIC level “A” or PharmGKB level “A1” evidence criteria.

We characterized “new” patients to evaluate the frequency and complexity of PGx drug ordering among individuals presumed naïve to PGx testing at the start of the extraction period. A “new” patient was defined as having no prior PGx drug history and at least one encounter occurring one year before their first PGx drug order.

As performed previously, the frequency and duration of drug orders were analyzed using an “ordering episode” heuristic [15]. An episode was defined as all orders for the same drug occurring within a year and a half of each other. This approach grouped recurring annual or more frequent orders into a single episode. Because computational date calculations are precise but real-world scheduling can vary, we applied an additional 0.5-year buffer to account for this variability. For patients receiving multiple PGx medications, we determined an “ordering interval,” defined as the number of days between the first prescription of the initial PGx drug and the earliest prescription of the final PGx drug during the study period. This was a consistent approach from the 2015–2019 study [15].

## 3. Results

Demographic Comparison of Patients with Clinical Encounters, Medication Prescriptions, and CPIC-Listed Drug Orders, 2020–2024: Table 1 provides a summary of the demographic characteristics of 928,291 patient charts analyzed from the EMR over the five-year period from 2020 to 2024. Females were more represented (54.5%) than males (45.5%). Based on these 928,291 encounters, there were 709,673 medication orders processed. A total of 416,621 (44.8%) patients had medication orders represented by the 75 CPIC drugs.

### 3.1. Comparison of CPIC Drug Ordering Characteristics 2020–2024

As shown in Table 2, CPIC drug ordering characteristics are organized according to CPIC guidelines, with counts based on unique patients. Ondansetron was the most frequently prescribed medication (255,153 orders in 61.0% of all CPIC patients) with a 26.9% change from 2015 to 2019 [15]. This was followed by non-steroidal anti-inflammatory drugs (NSAIDs) (ibuprofen, meloxicam, celecoxib, piroxicam, and flurbiprofen), statins (rosuvastatin, pitavastatin, fluvastatin, simvastatin, lovastatin, atorvastatin, and pravastatin), opioids (hydrocodone, tramadol, and codeine), proton pump inhibitors (PPIs) (pantoprazole, dexlansoprazole, lansoprazole, and omeprazole), and selective serotonin reuptake inhibitors (SSRIs) (escitalopram, paroxetine, venlafaxine, fluvoxamine, sertraline, vortioxetine, and citalopram). Of note, some of these medications, including ondansetron or NSAIDs, may have been prescribed within an order set or with directions to be taken as needed, which does not guarantee that the patient used the medication. However, with the medication being ordered, this allows for the use of the medication and therefore shows the potential benefit of PGx testing.

Table 2 shows the percent change between medication prescribed as per CPIC guidelines between the 2015–2019 and 2020–2024 groups. This highlights the prescribing trends during this time period. The medication with the greatest percentage increase in prescribing was atomoxetine, prescribed only 809 times during the earlier timeframe compared to 1854 times during the later (129.2% increase).

### 3.2. Combinations of CPIC Drugs Ordered 2020–2024

The demographic characteristics of the 2020–2024 cohort in Table 3 that used a CPIC drug are described, with the majority being female (57.1%) and White (74.5%), followed by other races of Black (17.1%) and Asian (2.3%). The most common ethnicity identified was non-Hispanic (93.6%).

Of the 75 total drugs with CPIC guidelines assessed in the data set, only 63 had orders identified in the EHR data. Overall, of these 63 medications ordered, 57 were ordered singly at least once (the number in the first row of Table 3), and 6 were only ever ordered in combination, hence the apparent discrepancy between 63 drugs but only 57 distinct single drug orders in Table 3.

As shown in Table 3, from 2020 to 2024, 156,578 patients (37.4%) were prescribed at least one CPIC medication, encompassing 57 distinct drug combinations. Among these, 100,396 patients (24.0%) were prescribed two CPIC medications, and 63,363 patients (15.2%) were prescribed three CPIC medications. In comparison, during 2015 to 2019, 133,380 patients (39.7%) were prescribed at least one CPIC medication with the same 57 drug combinations; 84,334 patients (25.1%) were prescribed two CPIC medications, and 51,357 patients (15.3%) were prescribed three CPIC medications.

## 4. Discussion

The 928,291 patients identified between the 2020–2024 extraction period was more than what was observed for the 2015–2019 timeframe (845,518). The results from this study show that 416,621 (44.8%) were prescribed a CPIC-actionable medication within the academic medical center between the years 2020 and 2024.

A total of 335,849 patient records were reviewed from 2015 to 2019. Among these, 133,380 patients (39.7%) were prescribed at least one CPIC medication, representing 47 distinct drug combinations. Approximately one-quarter of patients (84,334, 25.1%) received two CPIC medications, and 51,357 (15.3%) received three, accounting for 80.1% of patients prescribed between one and three CPIC medications during this period [15]. In comparison, the 2020–2024 analysis identified 418,144 unique patients, of whom 156,578 (37.4%) were prescribed at least one CPIC medication, covering 57 distinct drug combinations. Additionally, 100,396 patients (24.0%) were prescribed two CPIC medications, and 63,363 patients (15.2%) were prescribed three CPIC medications. Overall, three-quarters (76.6%) of patients were prescribed between one and three CPIC medications during this period, 2020–2024. Patients who were prescribed three or less CPIC drugs had the same pattern in both collection periods: 80.1% (2015–2019) and 76.6% (2020–2024) [15]. Given that a significant number of patients were prescribed three or less CPIC drugs, it would help a healthcare system project potential volume of patients if PGx services were to be offered.

The number of medications recommended for PGx testing and monitoring continues to increase due, in part, to the continued growth of research evidence [5]. This is, in turn, reflected in the growing list of drug–gene pairs added by the scientific community to CPIC guidelines, which did increase over the specified time periods observed in these two studies. This growth reflects advancements in PGx research, improvements in knowledge of drug–gene interactions, and broader adoption of precision medicine approaches across different specialties and health systems [17]. As prescribing patterns change and more medications are added to CPIC guidelines, it is crucial that pharmacists and providers are equipped to manage this added complexity.

Prescribing patterns of certain medications were different between the different timeframes, with increasing and decreasing frequencies. While this study did not identify the discipline of the prescribers, it would be reasonable to assume that the 19 medical specialties identified previously, with the highest frequencies observed from the departments of anesthesiology, oncology, internal medicine, and family practice, would be similar, as the medical center and EHR remained the same [15]. This is important if an organization determines it wants to offer PGx services and in what departments or disciplines it is most likely to prescribe medications identified by the CPIC.

Paradoxically, while the number of CPIC-guided medications has increased, this is not always reflective in clinical practice and thus the data. Some older medications that have CPIC guidelines may be prescribed less due to advances in other therapeutic categories and recommended clinical guidelines. Warfarin is an example of this with the gene VCORC1, and other anticoagulant agents can be substituted that do not fall under CPIC guidelines.

The clinical significance of PGx testing lies in its ability to use evidence-based guidelines, such as those from the CPIC, to translate patient-specific genotypes into actionable phenotypes. This process enables clinicians to optimize medication selection, avoid harmful drug–gene interactions, and adjust dosing appropriately [2,7]. PGx-guided prescribing can enhance patient safety, improve therapeutic outcomes, and, in some cases, reduce overall healthcare costs. Integrating PGx data into EMRs is critical for enabling timely decision support for clinicians and ensuring seamless implementation in routine practice [13].

With multiple prescribers encountered in the routine care of patients, having a single discipline overseeing PGx services makes sense from a resource and workflow standpoint, as well as based on economic perspectives for health systems. Pharmacists are well positioned to lead PGx services through precision medicine efforts as well as contribute to individual’s health outcomes, given their unique pharmacotherapy knowledge related to a wide array of medications.

This study has limitations. It included patients and prescribers from a single academic medical center, so regional and national prescribing trends were not assessed. The analysis did not determine whether any PGx tests had been ordered, as this was beyond the scope of the project. Patients included in this analysis may have been assessed in the 2015–2019 cohort; however, each encounter was different so it is important to continue to emphasize the utility of genetic testing throughout a patient’s lifetime [15].

Additionally, all comparisons in Table 2 relied on raw prescribing counts for distinct patients and did not adjust for changes in total prescriptions or patient volume between the periods 2015–219 and 2020–2024, limiting the interpretation between the two time periods. However, the top five classes of medications remained the same in the two periods (ondansetron, statins, proton-pump inhibitors, codeine, and NSAIDs). Such information is useful when assessing the implementation of a PGx service.

Another limitation is that some drugs are prescribed less, in part due to the lack of knowledge associated with pharmacogenomics and education provided to prescribers on the risk of certain classes of medications. For example, in the codeine-related products, a reduction of 21.3% was observed in codeine, tramadol, and hydrocodone at this institution. This reduction in these medications may, in part, be explained by external and internal guidelines on prescribing analgesics for acute pain management.

Also, it is recognized that between 2020 and 2024, the COVID-19 pandemic led to a change in healthcare utilization. Clinicians needed to manage both acute infections and complications in patients with many comorbidities, leading to increased prescription and over-the-counter medications used. The use of medications with pharmacogenomic considerations became increasingly more prominent as CPIC-guided drugs—such as analgesics (opioid and non-opioid) and antidepressants—were frequently used to treat underlying conditions or symptoms that coexisted with COVID-19 [18,19]. The analysis of the impact of the pandemic was beyond the scope of this study.

## 5. Conclusions

Given the consistency observed where about three-quarters of patients over the ten-year period had between one and three CPIC drugs prescribed, as well as the common medication classes identified, leveraging EMR data provides an effective means to quantify patients and the medication involved who may be eligible for PGx testing. This analysis and the findings may help healthcare organizations as they begin to assess the implementation of PGx services as more precision medicine platforms are adopted.

## Figures and Tables

**Table 1:** Demographics of patients with clinical encounters, medication orders, and CPIC-listed drug orders for 2020–2024.

	Patients with Encounters			Patients with Medication Orders			Patients with CPIC Drug Orders		
Sex	Patients	Median Age	IQR	Patients	Median Age	IQR	Patients	Median Age	IQR
Female	505,921	47	(30–66)	391,347	47	(30–66)	237,825	51.0	(34–69)
Male	422,192	47	(29–66)	318,307	48	(30–66)	178,790	56.0	(38–70)
Other/Unknown	178	67	(37–103)	19	43	(29–61)	6	33.5	(22–40)

**Table 2:** CPIC drug ordering characteristics for 2015–2019 [15] compared to 2020–2024, grouped by CPIC guidelines and patients.

		Distinct Patients			
Guideline Abbreviation	Guideline Drugs	2015–2019	2020–2024	Percent (%) Change	% of All CPIC Patients 2020–2024
Abacavir	abacavir	337	177	−47.5%	0.0%
Allopurinol	allopurinol	11,441	14,815	29.5%	3.5%
Atazanavir	atazanavir	87	13	−85.1%	0.0%
Atomoxetine	atomoxetine	809	1854	129.2%	0.4%
Carbamazepine	carbamazepine and oxcarbazepine	2657	3181	19.7%	0.8%
Clopidogrel	clopidogrel	14,957	17,425	16.5%	4.2%
Codeine	codeine, tramadol, and hydrocodone	137,499	108,261	−21.3%	25.9%
Efavirenz	efavirenz	185	29	−84.3%	0.0%
Fluoropyrimidines	fluorouracil and capecitabine	3975	5498	38.3%	1.3%
Glucose-6-Phosphate Dehydrogenase (G6PD)	dapsone, methylene blue, pegloticase, tafenoquine, primaquine, nitrofurantoin, and rasburicase	27,894	39,957	43.2%	9.6%
Ivacaftor	ivacaftor	98	152	55.1%	0.0%
NSAIDs	ibuprofen, piroxicam, meloxicam, flurbiprofen, and celecoxib	122,530	139,289	13.7%	33.3%
Ondansetron	ondansetron	201,054	255,153	26.9%	61.0%
PEG *	peginterferon alfa-2a and peginterferon alfa-2b	20	31	55.0%	0.0%
Phenytoin	phenytoin and fosphenytoin	2540	1424	−43.9%	0.3%
PPI	pantoprazole, dexlansoprazole, lansoprazole, and omeprazole	91,740	119,285	30.0%	28.5%
Ryanodine Receptor 1 (RYR1)	isoflurane, succinylcholine, sevoflurane, desflurane, and enflurane	21,373	19,798	−7.4%	4.7%
SSRI	escitalopram, paroxetine, venlafaxine, fluvoxamine, sertraline, vortioxetine, and citalopram	61,303	84,913	38.5%	20.3%
Statins	rosuvastatin, pitavastatin, fluvastatin, simvastatin, lovastatin, atorvastatin, and pravastatin	88,600	126,295	42.5%	30.2%
Tacrolimus	tacrolimus	4741	7705	62.5%	1.8%
Tamoxifen	tamoxifen	1540	1772	15.1%	0.4%
Thiopurines	thioguanine, azathioprine, and mercaptopurine	2734	2297	−16.0%	0.5%
Tricyclic [Antidepressants]	doxepin, imipramine, trimipramine, nortriptyline, clomipramine, desipramine, and amitriptyline	14,304	14,415	0.8%	3.4%
Voriconazole	voriconazole	1140	929	−18.5%	0.2%
Warfarin	warfarin	16,006	11,118	−30.5%	2.7%

* PEG = Peginterferon alpha-based regimens.

**Table 3:** Comparison of combinations of CPIC drugs ordered in 2015–2019 [15] vs. 2020–2024.

					Interval Days ^1^					
Total Number of CPIC Drugs	Unique Patients		Distinct Drug Combinations		Median		IQR		99th Percentile	
	2015–2019 N = 335,849	2020–2024 N = 418,144	2020–2024	2015–2019	2015–2019	2020–2024	2015–2019	2020–2024	2015–2019	2020–2024
1	133,380 39.7%	156,578 37.4%	57 ^2^	47	NA	NA	NA	NA	NA	NA
2	84,334 25.1%	100,396 24.0%	788	593	17	4	(0–343)	(0–254)	1548	1530
3	51,357 15.3%	63,363 15.2%	3119	2090	224	172	(3–737)	(2–661)	1668	1647
4	29,628 8.8%	40,191 9.6%	6150	3819	450	457	(50–991)	(86–976)	1721	1709
5	17,560 5.2%	24,746 5.9%	8085	4678	672	694	(204–1163)	(250–1159)	1747	1746
6	9772 2.9%	15,105 3.6%	8125	4479	850	875	(357–1298)	(433–1299)	1759	1765
7	5197 1.5%	8651 2.1%	6331	3457	993	1035	(526–1374)	(617–1398)	1770	1769
8	2600 0.8%	4717 1.1%	4095	2095	1129	1153	(716–1444)	(762–1473)	1773	1779
9	1233 0.4%	2427 0.6%	2302	1133	1227	1272	(843–1504)	(902–1529)	1784	1791
10 or more	792 0.2%	1970 0.5%	1946	776	1337	1317	(951–1569)	(985–1563)	1798	1792

^1^ Ordering interval is defined as the number of days between the first order of the first drug and the earliest order of last drug in a combination for a patient. ^2^ In total, 47 and 57distinct CPIC drugs were ordered singly for patients who received no other CPIC drugs during the 2015–2029 and 2020–2024 study periods, respectively. NA = Non Applicable.

## Data Availability

The data presented in this study are available on request from the corresponding author due to privacy or ethical restrictions.

## Abbreviations

The following abbreviations are used in this manuscript:

EMR	Electronic Medical Record
CPIC	Clinical Pharmacogenetics Implementation Consortium
PGx	Pharmacogenomics
